# Understanding the developmental well-being of children from refugee backgrounds in British Columbia, Canada: A population-level mixed methods approach

**DOI:** 10.23889/ijpds.v9i5.2562

**Published:** 2024-09-18

**Authors:** Monique Gagné Petteni, Anne Gadermann, Martin Guhn, Brenda Poon, Magdalena Janus, Benjamin Edwards

**Affiliations:** 1 University of British Columbia; 2 McMaster University; 3 Australian National University

## Objectives and Approach

This study utilized an explanatory sequential mixed methods approach to investigate the developmental well-being of children from refugee backgrounds in British Columbia (BC), Canada. Objective 1 (quantitative) leveraged population-level, government administrative data individually linked to a province-wide, routinely collected, teacher-reported measure of children’s development in kindergarten (the Early Development Instrument; EDI) to examine developmental outcomes across five domains for children identified as first-generation refugees (N=770), first-generation immigrants (N=7875), and non-migrants (N=199,186). In Objective 2 (qualitative), the population-level EDI results were brought to focus groups with BC educators and settlement workers (N=7) who work closely with children from refugee backgrounds to further corroborate, expand, and elaborate on the findings.

## Results

The A series of multiple linear regression models; adjusted for age, sex, and English Language Learner status showed that first-generation refugee status was significantly predictive of lower EDI scores in the areas of language & cognitive development, communication & generation knowledge, social competence, emotional maturity, and physical health & well-being. Focus group results corroborated the quantitative findings, added critical complexity/context (e.g., impacts of trauma), and identified important policy-oriented levers (e.g., early, accessible assessments and supports).

## Conclusions

The study provided an understanding of the population-level developmental well-being of children from refugee backgrounds in BC, framed by rich, contextualized, and actionable knowledge from focus groups.

## Implications

Showcasing the combined breadth and depth of using a mixed methods approach, how we can best support the developmental challenges and build upon the strengths of children from refugee backgrounds will be discussed.

